# Dual targeting of retinoid X receptor and histone deacetylase with DW22 as a novel antitumor approach

**DOI:** 10.18632/oncotarget.3149

**Published:** 2015-02-05

**Authors:** Lihui Wang, Guoliang Chen, Kang Chen, Yong Ren, Huahuan Li, Xiaorui Jiang, Lina Jia, Shiyuan Fu, Yi Li, Xinwei Liu, Shuang Wang, Jingyu Yang, Chunfu Wu

**Affiliations:** ^1^ Department of Pharmacology, Shenyang Pharmaceutical University, Shenyang, P.R. China; ^2^ Benxi Institute of Pharmaceutical Research, Shenyang Pharmaceutical University, Benxi, P.R. China; ^3^ Key Laboratory of Structure-Based Drugs Design & Discovery of Ministry of Education, Shenyang Pharmaceutical University, Shenyang, P.R. China; ^4^ Department of Pathology, Wuhan General Hospital of Guangzhou Command, People's Liberation Army, Wuhan, P.R. China

**Keywords:** Retinoid X receptor, Histone deacetylase, DW22, anti-tumor

## Abstract

Retinoid X receptor (RXR) and Histone deacetylase (HDAC) are considered important targets for cancer therapy due to their crucial roles in genetic or epigenetic regulations of cancer development and progression. Here, we evaluated the potential of dual targeting of RXR and HDAC using DW22 as a novel therapeutic approach to cancer treatment. We found that the co-expression of RXR-α and HDAC1 was frequently appeared in lung cancer and breast cancer tissues and cell lines. RXR was activated by DW22 in RXRα and HDAC1 overexpressed A549 and MDA-MB-435 cell lines. Meanwhile, DW22 inhibited the activity of HDAC by decreasing its expression in A549 and MDA-MB-435 cell lines, but not in RXRα and HDAC1 deficient cell lines. Moreover, DW22 suppressed cell growth, induced cell differentiation, prompted cell apoptosis and arrested cell cycle in A549, MDA-MB-435 or HL60 cell lines. Treatment human umbilical vascular endothelial cells (HUVECs) with DW22 suppressed migration, invasion and tube formation through decreasing VEGF expression. The up-regulation of Ac-H3 and p21, and down-regulation of VEGF caused by DW22 was markedly attenuated by silencing of HDAC1. Furthermore, knockdown of RXRα by siRNA completely blocked DW22-induced cell differentiation, but partially attenuated DW22-caused inhibition of cell proliferation, induction of cell apoptosis, and suppression of cell migration, invasion and tube formation. Moreover, intravenous administration of DW22 significantly retarded tumor growth of A549 and MDA-MB-435 xenograft mice models, and induced no substantial weight loss and gross toxicity. In addition, DW22 also reduced cell proliferation, angiogenesis, and induced cell apoptosis *in vivo*. Collectively, our data demonstrates that dual targeting of RXR and HDAC using DW22 possesses pleiotropic antitumor activities both *in vitro* and *in vivo*, providing a novel therapeutic approach for cancer treatment.

## INTRODUCTION

The hallmarks of cancer comprise multiple biological capabilities, including sustaining proliferative signaling, evading growth suppressors, resisting cell death, enabling replicative immortality, inducing angiogenesis, and activating invasion and metastasis [1–3]. Therefore, the multi-target therapeutic approach to comprehensively inhibit the biological capabilities of tumors is considered as the ideal therapy strategy [4, 5].

Retinoid X receptor (RXR), a well-known molecular target in several human tumors [6], executes its biological functions with retinoid acid receptor (RAR) to modulate the transcriptional activity of retinoid receptor target genes associated with cell growth and differentiation [6]. Bexarotene, a small molecule RXR agonist, was successfully tested in cutaneous T-cell lymphoma (CTCL) and had been approved by FDA [7], indicating the potential of RXR as an anti-cancer target. Recently, several clinical trials were ongoing to assess the potential of Bexarotene for treatment of other cancers, such as lung cancer [8, 9] and breast cancer [10], but failed to show clinical benefit. These data suggest that targeting a single RXR in these cancers may not be efficient enough for successful treatment of these deadly diseases.

The mammalian target of histone deacetylase (HDAC) was intensively studied in a multitude of human tumor entities over the past couple of years [11]. HDAC, that deacetylates lysines on core histones and other cellular proteins, plays the crucial roles in the epigenetic regulation of gene transcription and controlling cellular functions, such as cell-cycle, terminal differentiation, apoptosis, migration, invasion and angiogenesis [12]. Two HDAC inhibitors, SAHA (vorinostat) and FK228 (romidepsin), have been approved by FDA for the treatment of CTCL [13], indicating that HDAC is an important target for tumor therapy.

Recent research has shown that the combination of RXR agonist and HDAC inhibitor synergistically blocked the growth of tumors in preclinical and clinical studies [14, 15], which represents a possible molecular link between the RXR and the HDAC. In fact, a previous study also demonstrated there was an inter-regulation between RXR and HDAC. Li Y *et al* demonstrated that RXR agonist Bexarotene causes the recruitment of HDAC to the target gene's promoter and then resulting in transcriptional repression [16], suggested that there might be an opposite relationship between RXR activation and HDAC inhibition. Taken together, we hypothesis it might be an ideal anti-tumor approach by activating RXR simultaneously inhibiting HDAC. In our previous study, we identified a compound, DW22, which could activate RXR and inhibit HDAC in cancer cells, and also demonstrated the efficacy as an antitumor agent in representative cancer cell lines and drug-resistant cancer cell lines [17]. Here, we further demonstrate that dual targeting RXR and HDAC using DW22 possesses pleiotropic antitumor activities *in vitro* and *in vivo*, providing a novel therapeutic approach for cancer treatment.

## RESULTS

### Dual overexpression of RXR and HDAC is prevalent in human tumors

**Figure 1:**
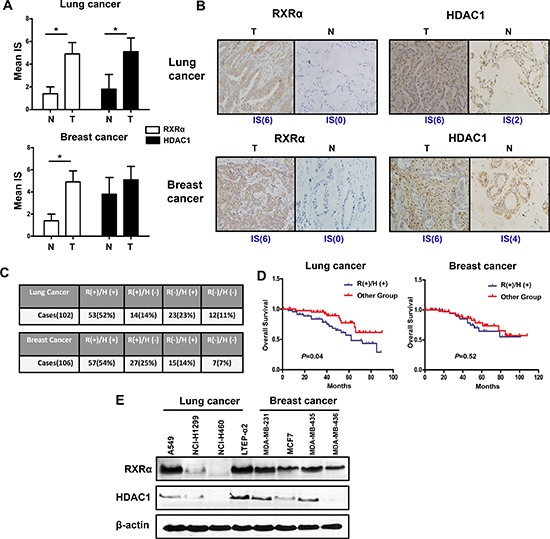
RXRα and HDAC1 are highly expressed in human breast and lung cancer tissues and cell lines **(A)** The expression of RXRα and HDAC1 in lung tissues (Left panel, number of adjacent normal tissues = 12) and breast tissues (Right panel, number of adjacent normal tissues = 15). N(normal tissue), T(tumor tissue), IS(immunoreactive score). *p < 0.05 compare with normal tissue group. **(B)** The expression of RXRα and HDAC1 in representative breast and lung cancer tissues. Figures magnified 400x. **(C)** The co-expression rate of RXRα and HDAC1 in lung and breast cancer tissues. A sample is defined as RXRα or HDAC1 + if it has an IS ≥ 2. R(RXRα), H(HDAC1). **(D)** Overall survival according to co-expression of RXRα and HDAC1 in lung cancer and breast cancer. **(E)** The expressions of RXRα and HDAC1 in lung cancer and breast cancer cell lines were measured by western blotting. β-actin expression was used as a loading control (RXRα, MW 53 kD; HDAC1, MW 62 kD; β-actin, MW 43 kD).

To validate our proposed concept of anti-tumor approach by dual RXR/HDAC targeting, it was critical to analyze RXR and HDAC expression in human tumors. Using immunohistochemistry method, we detected RXR-α and HDAC1, which are mainly functional subtype of RXR and HDAC respectively, in human lung and breast tissues. As shown in Figure 1A and 1B, RXR-α was expressed, especially in the cytoplasm, at a higher level in lung cancer when compared with that in adjacent non-cancerous (normal) tissues (Figure 1A and 1B). HDAC1 showed a similar pattern to RXR-α (Figure 1A and 1B). Interestingly, we detected that 52% lung cancers expressed both proteins in high level (Figure 1C). Consistent with previous report [18], the level of RXR-α was lower in normal breast tissues. However, RXR-α was markedly enhanced in breast cancer tissues (Figure 1A and 1B). In contrast to lung tissue, there was no significant difference of HDAC1 expression between breast tumor tissues and normal tissues (Figure 1A and 1B). Importantly, a relative higher co-expressed rate of RXR-α and HDAC1 (54%) was also found in breast cancer tissues (Figure 1C). Furthermore, co-expression of RXR-α and HDAC1 correlated with poor prognosis than either single- or double-negative RXR-α and HDAC1 groups in lung cancer, but not in breast cancer (Figure 1D). In accord with results of tumor tissues, most of RXR-α overexpressed cell lines also expressed HDAC1 at a relative high level in both lung cancer and breast cancer cell lines (Figure 1E). Taken together, these results strongly support the feasibility of proposed RXR/HDAC targeted therapy for human tumors.

### DW22 activates RXR and inhibits HDAC in human cancer cell lines

**Figure 2:**
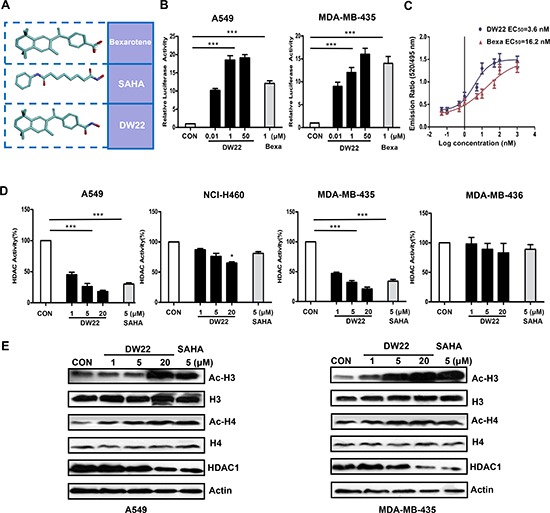
The effects of DW22 on RXR activation and HDAC inhibition **(A)** 3D structure of Bexarotene, SAHA and DW22. **(B)** *In vitro* activation of RXR by DW22 in different concentrations (10 nM, 1 μM, and 50 μM). **(C)** Lanthascreen TR-FRET assay, demonstrating that DW22 increased the binding of the RXRα to coactivator peptide PGC1α *in vitro*. **(D)** *In vitro* inhibition of HDAC by DW22 in different concentrations (1 μM, 5 μM, and 20 μM). **(E)** Acetylated histone-3(Ac-H3, MW 17 kD), histone-3(H3, MW 17 kD), acetylated histone-4(Ac-H4, MW 10 kD), histone-4(H4, MW 11 kD), and HDAC1(MW 62 kD) were measured in A549 and MDA-MB-435 cell lines after DW22 (1, 5, 20 μM) treated for 48 h. β-actin expression was used as a loading control. All error bars are s.e.m. ***p < 0.005 compare with control group, *p < 0.05 compare with control group.

DW22 was identified as a compound dual-targeting of RXR and HDAC [17] (See Figure 2A). Here, we examined the effect of DW22 on RXR activation using cell-based transactivation assays in RXR-α overexpressed cell lines A549 and MDA-MB-435. It was showed that treatment of A549 or MDA-MB-435 cells with DW22 significantly activated RXR reporter in a concentration-dependent manner (Figure 2B). As a positive control, Bexarotene (1 μM) treatment also resulted in an activation of RXR. To explore the activation mechanism, we detected the expression level of RXRα after treatment with DW22 in both cell lines. Western blot analysis data showed that either DW22 or Bexarotene had no effect on the expression of RXRα (Data not shown). These results demonstrate that DW22 can activate RXRα irrespective of its expression in A549 or MDA-MB-435 cells. The observations described above raise the possibility that DW22 might be an agonist of RXR. To test this hypothesis we examined the effect of DW22 on RXRα coactivator interaction *in vitro* by TR-FRET. In this assay, the interaction of the RXRα (indirectly labeled by terbium) with the coactivator peptide PGC1α (labeled with fluorescein) was detected. As shown in Figure 2C, DW22 treatment resulted in an enhanced binding of the RXRα to coactivator peptide PGC1α (EC_50_ = 3.6 nmol/L) compared to the well-studied RXR agonist, Bexarotene (EC_50_ = 16.2 nmol/L). These results suggest that DW22 is a ligand and an agonist of RXRα.

We subsequently tested the HDAC inhibition activity of DW22 in both HDAC1 overexpressed cell lines (A549 and MDA-MB-435) and paired HDAC1 deficient cell lines (NCI-H460 and MDA-MB-436). Our data indicated that DW22 exhibited markedly HDAC inhibitory activity among all measured concentrations in A549 and MDA-MB-435 cells, but not in NCI-H460 and MDA-MB-436 cells (Figure 2D). In order to verify the HDAC inhibition of DW22, the effects of DW22 on the level of acetylated histones were examined by western blot analysis using the specific antibodies against acetylated H3 and H4. As a result, DW22 significantly increased the levels of acetylated H3 and H4 (Figure 2E) in a concentration-dependent manner in A549 and MDA-MB-435 cells. However, DW22-caused increase of acetylated H3 and H4 were significant attenuated in NCI-H460 and MDA-MB-436 cells when compared to those in A549 and MDA-MB-435 cells (Supplementary Figure 1). The crucial role of HDAC inhibition in acetylation of histones was confirmed by silencing HDAC1 in A549 cells. The increase of acetylated H3 induced by DW22 was blocked by specific HDAC1 siRNA (Supplementary Figure 2). In addition, we also detected the effects of DW22 and SAHA on the expression level of HDAC1. The data indicated that, similar to SAHA, DW22 decreased the expression of HDAC1 in A549 and MDA-MB-435 cell lines (Figure 2E). Taken together, these results suggest that DW22 could inhibit HDAC activity through downregulating its expression.

### DW22 inhibits cell proliferation and induces cell differentiation in human cancer cells

**Figure 3:**
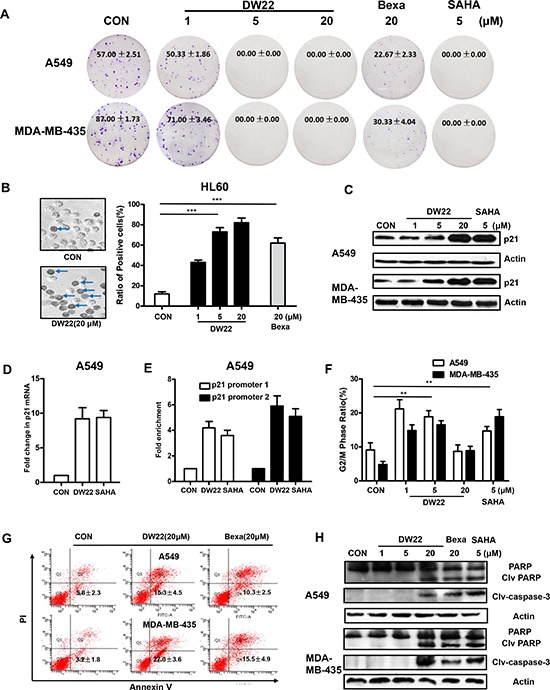
The effects of DW22 on colony formation, cell differentiation, p21 expression, cell cycle and apoptosis **(A)** Colony forming ability of A549 and MDA-MB-435 cells was inhibited by DW22 (1 μM, 5 μM, and 20 μM) treatment for 7 days. **(B)** The differentiation numbers of HL-60 cells were increased by DW22 (1 μM, 5 μM, and 20 μM) treatment for 4 days. The blue arrows indicate differentiated cells (positive cells). Quantification of the effect of DW22 on cell differentiation is shown column diagram. **(C)** p21^WAF1/CIP1^ protein was measured by western blot analyses in A549 and MDA-MB-435 cell lines after DW22 (1, 5, 20 μM) treated for 48 h. β-actin expression was used as a loading control. **(D)** The mRNAs were analyzed for p21 levels by quantitative PCR using specific primers as described in Materials and Methods. GAPDH was used as control. DW22(20 μM); SAHA(5 μM). **(E)** ChIP assays confirmed the binding of acetylated histone-3 to the promoter regions upstream of *p21* gene. Promoter 1 located in −742 to −488 upstream of *p21* gene; promoter 2 located in −2894 to −1753 upstream of *p21* gene. DW22(20 μM); SAHA(5 μM). The G_2_/M phase **(F)** and apoptotic cells **(G)** were assessed by FACS analyses in A549 and MDA-MB-435 cell lines after DW22 (1, 5, 20 μM) treated for 48 h. **(H)** The apoptosis-related proteins (clv-caspase-3 and PARP) were assessed by western blot analyses in A549 and MDA-MB-435 cell lines after DW22 (1, 5, 20 μM) treated for 48 h. β-actin expression was used as a loading control. Clv-caspase-3, MW 17 kD; PARP, MW 116 kD; clv-PARP, MW 89 kD. All error bars are s.e.m. *p < 0.05 compare with control group, **p < 0.01 compare with control group, ***p < 0.005 compare with control group.

To verify the importance of RXR and HDAC, we next investigated the anti-proliferative effect of DW22 on both overexpressed targets cell lines and deficient targets cell lines using MTT method. As shown in Table 1, DW22 treatment exhibited an anti-proliferative effects on overexpressed targets cell lines (A549 and MDA-MB-435) with a mean IC_50_ of 6.8 μM, which is obviously lower than that of deficient targets cell lines (NCI-H460 and MDA-MB-436, mean IC_50_ > 54.1 μM). Similar tendency was also observed in SAHA or Bexarotene treated cells. We further tested the ability of DW22 to inhibit cell proliferation in A549 and MDA-MB-435 cell lines using clonogenicity assay. The results revealed that DW22 inhibited cell proliferation of both cell lines in concentration-dependent manner, and the inhibitory activity of DW22 was comparable with SAHA and far more potently than Bexarotene (Figure 3A).

**Table 1:** Antiproliferative activities of DW22, SAHA and Bexarotene

Cmpnd	IC_50_(μM)						
	A549	MDA-MB-435	Mean	NCI-H460	MDA-MB-436	Mean
**DW-22**	4.9 ± 2.1	8.8 ± 2.6	**6.8**	8.1 ± 0.5	>100	**>54.1**
**SAHA**	11.5 ± 2.0	41.2 ± 6.1	**26.5**	97.8 ± 11.8	>100	**>98.9**
**Bexarotene**	25.7 ± 5.3	12.5 ± 3.6	**19.2**	22.8 ± 1.8	>100	**>61.4**

To determine the effects of DW22 on cell differentiation, HL-60 cells were treated with different concentrations of the compounds, and the extent of myeloid cell differentiation was assessed by the NBT reduction assay. As shown in Figure 3B, the addition of DW22 to cultures resulted in a marked concentration-dependent increase in the degree of differentiation. Confirmed the RXR activation data, in the same concentration (20 μM), DW22 displayed an enhanced ability to induce differentiation as compared with Bexarotene.

### DW22 induces up-regulation of p21^WAF1/CIP1^, arrests cell cycle and causes apoptosis in human cancer cells

It has been demonstrated that the induction of p21^WAF1/CIP1^, a cyclin-dependent kinase inhibitor, is the result of HDAC inhibitor-mediated histones acetylation [19]. Therefore, we further measured the levels of p21 ^WAF1/CIP1^ to verify the effect of DW22 on the HDAC. The results showed that DW22 could increase the expression of p21^WAF1/CIP1^ in a concentration-dependent manner in A549 and MDA-MB-435 cell lines (Figure 3C). In addition, p21 mRNA was also upregulated by DW22 in A549 cells (Figure 3D). To correlate p21 up-regulation and localized chromatin organization, the binding of acetylated histone H3 was analyzed at the p21 promoter using ChIP assay. As shown in Figure 3E, two reported regions (−742 to −488 and −2894 to −1753) showed a 3–6 folds enrichment of the acetylated histones H3 in DW22 treated A549 cells [20]. The role of HDAC inhibition in p21 induction was confirmed by silencing HDAC1 in A549 cells. The DW22-induced increase of p21 was reversed by specific HDAC1 siRNA (Supplementary Figure 3).

The induction of p21 by DW22 was well confirmed by cell arrest data in A549 and MDA-MB-435 cell lines. Cell cycle analysis results indicated that DW22, at the concentrations of 1 and 5 μM, induced a significant arrest in the G_2_-M phase (Figure 3F). Interestingly, we found that DW22, at a relative higher concentration (20 μM), could not induce an obvious arrest, but lead to a proportional increase in SubG_1_ phase (data not shown) which is considered as apoptosis. Therefore, we next examined the effects of DW22 on cell apoptosis using AnnxinV/PI double staining methods. The data revealed that DW22 (20 μM) caused an increase in the percentage of apoptosis cells (Annexin V(+)/PI(–)) in A549 and MDA-MB-435 cell lines (Figure 3G). Compared with that of Bexarotene, DW22 exhibits an enhanced apoptosis-induced potential (Figure 3G). The above data was further confirmed by measuring the cleavages of PARP and Caspase-3 which are regarded as biomarkers of cell apoptosis. DW22 treatment at higher concentration (20 μM) resulted in an obvious increasing of the cleavages of PARP and Caspase-3 as compared to control, but there were no effects of DW22 at low concentrations (1 and 5 μM, Figure 3H). The targets related action of DW22 was confirmed using deficient targets cell lines (NCI-H460 and MDA-MB-436). The addition of DW22 could not lead to a marked cleavage of Caspase-3 in NCI-H460 and MDA-MB-436 cells (Supplementary Figure 4).

Taken together, these results demonstrate that the anti-proliferative action of DW22 to human cancer cells is accomplished through arrest of cell-cycle progression and induction of apoptosis.

### DW22 inhibits migration, invasion, and tube formation of endothelial cells through down-regulation of VEGF

**Figure 4:**
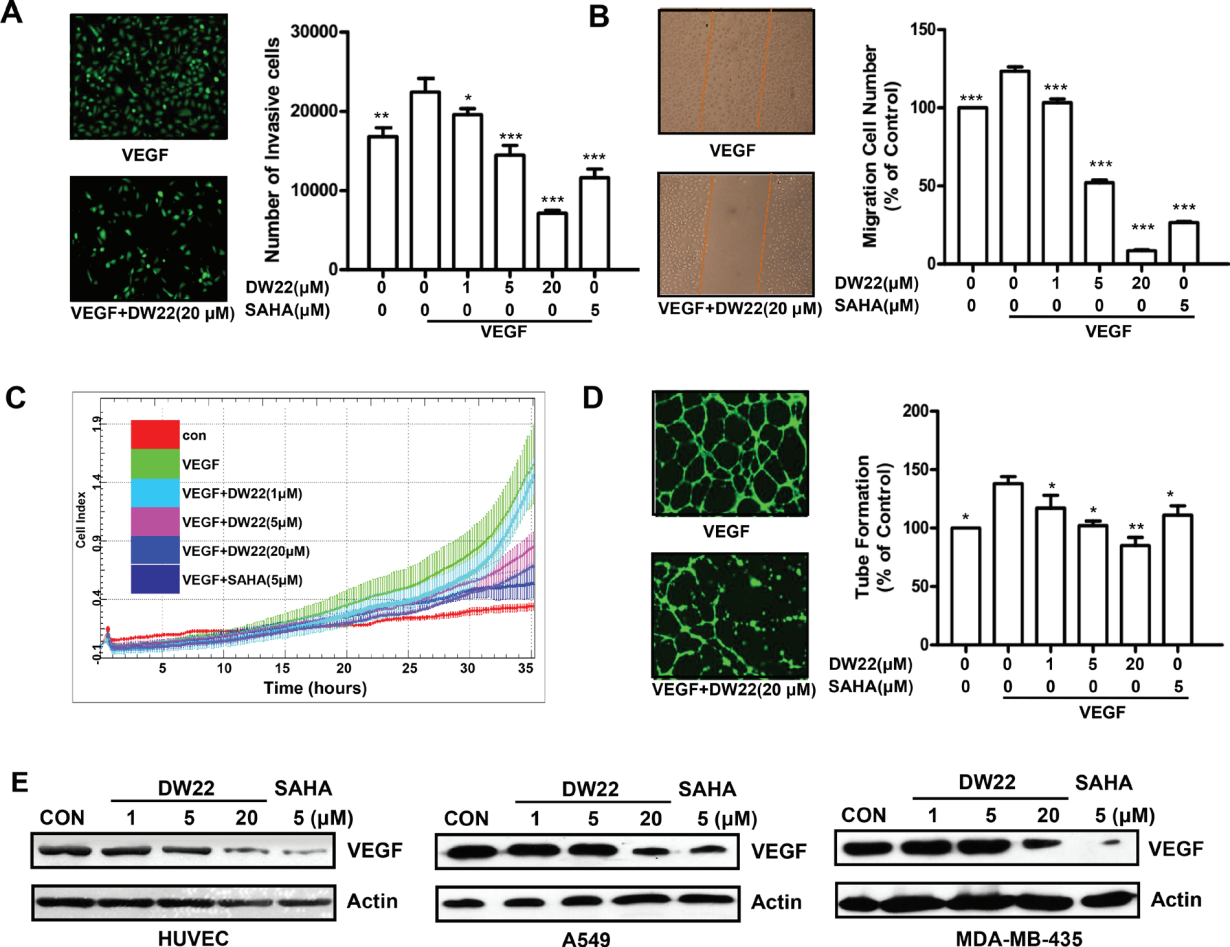
The effects of DW22 on VEGF-induced migration, invasion, and tubular structure formation of endothelial cells **(A)** HUVECs invasion was measured by transwell assay after DW22 (1, 5, 20 μM) treated for 12 h. **(B)** HUVECs migration was measured by wound-healing migration assay after DW22 (1, 5, 20 μM) treated for 12 h. **(C)** In the biosensor system, migrating cells were indicated by the cell index. Data were analyzed using RTCA software 1.2 (supplied with the instrument). **(D)** HUVECs tube formation was measured after DW22 (1, 5, 20 μM) treated for 12 h. The experiments were performed as described in Materials and Methods. **(E)** The VEGF expression was assessed by western blot analyses in HUVEC, A549 and MDA-MB-435 cell lines after DW22 (1, 5, 20 μM) treated for 24 h. β-actin expression was used as a loading control. VEGF, MW 43 kD; β-actin, MW 43 kD. All error bars are s.e.m. *p < 0.05 compare with VEGF control group, **p < 0.01 compare with VEGF control group, ***p < 0.005 compare with VEGF control group.

The migration, invasion and angiogenesis of endothelial cell are necessary for tumor growth and metastasis [21]. Thus, we assessed the effects of DW22 on VEGF-induced migration, invasion, and tube formation of HUVECs *in vitro*. In order to exclude the cytotoxicity of DW22, the viability of HUVECs after treatment with DW22 was firstly examined. DW22 treatment for 12 h caused a slight decrease in the percentage of metabolically viable HUVECs at 20 μM (Supplementary Figure 5). In addition, treatment with DW22 for 36 h only resulted in a 15% inhibition of cell viability (Data not shown). Therefore, the maximal concentration of 20 μM was chosen in the following experiments. The effects of DW22 on the chemotactic motility of HUVECs were measured by wound-healing migration assay, RTCA and Transwell cell invasion assay. It was showed that DW22 dramatically reduced VEGF-induced HUVECs invasion and migration (Figure 4A, 4B, and 4C). Both effects of DW22 were concentration dependent, with significant inhibition first occurring at 1 μM. In the same concentration (5 μM), DW22 displayed a relatively weak ability to inhibit migration of HUVECs as compared with SAHA.

Although angiogenesis is a very complex process, tube formation of endothelial cells is one of the key steps [22]. Next, we investigated how DW22 affects HUVEC tube formation using two-dimensioned Matrigel assay. As shown in Figure 4D, in the presence of VEGF, elongated and robust tube-like structures were formed. When treated with DW22, the formation of tubular structures was significantly blocked. Meanwhile, the results also revealed that the protein expressions of VEGF were downregulated by DW22 treatment (Figure 4E) in HUVECs, A549 and MDA-MB-435 cells, which suggested the impaired migration, invasion and tube formation ability might be present in DW22-treated solid tumor microenvironment through inhibiting VEGF expression.

To further explore the underlying mechanism of DW22-caused decrease of VEGF, we accessed the role of HDAC1 in this event. Silencing of HDAC1 by siRNA could markedly block the effects of DW22-caused VEGF decrease in A549 cells (Supplementary Figure 6). Previous study reported that HDAC1 down-regulation could result in an increase of KLF4 [23], which is considered as a repressor of VEGF [25]. Therefore, we hypothesis whether KLF4 is a directly regulator of VEGF down-regulation caused by DW22. Our data indicated that DW22 could increase the expression of KLF-4 in a concentration-dependent manner in HUVECs and A549 cells (Supplementary Figure 7). These results demonstrated that DW22 treatment resulted in HDAC inhibition, then led to up-regulation of KLF4, finally contributed to the down-regulation of VEGF.

### RXR silence attenuates DW22-mediated suppression of cell proliferation, migration, invasion, tube formation, and –induced cell differentiation and apoptosis

**Figure 5:**
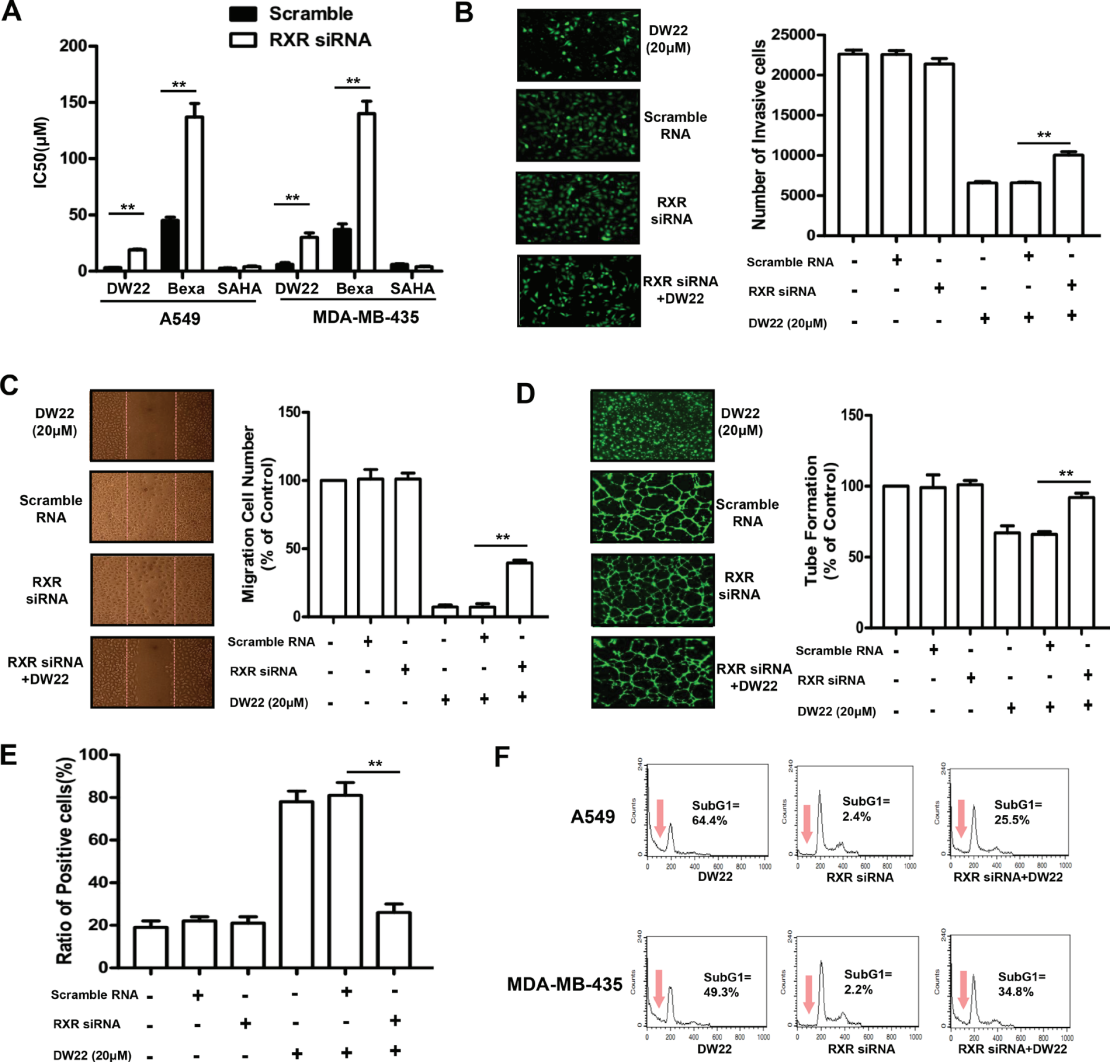
The effects of RXRα silence on DW22-caused anti-tumor activities **(A)** The cell viabilities were measured by MTT assay in RXRα silenced- and control A549 and MDA-MB-435 cells after DW22, Bexarotene and SAHA treatments for 48 h. The invasion **(B)**, tube formation **(C)**, and migration **(D)** of RXRα silenced and control HUVECs were assessed as described in Materials and Methods. The cells were treated with DW22 (20 μM) for 12 h. **(E)** The cell differentiation was measured by NBT assay in RXRα silenced- and control HL60 cells after DW22 (20 μM) treated for 4 days. **(F)** The cell apoptosis was analyzed by FACS in RXRα silenced- and control A549 and MDA-MB-435 cells after DW22 (20 μM) treated for 48 days. All error bars are s.e.m. *p < 0.05 compare with control group, **p < 0.01 compare with control group, ***p < 0.005 compare with control group.

To explore the role of the activation of RXR in DW22-mediated alterations of biological characteristics in human cancer or endothelial cells, we determined the effects of RXRα silence by specific siRNA on DW22-mediated suppression of cell proliferation, migration, invasion, tube formation, and –induced cell differentiation and apoptosis. Cells transfected with the scramble siRNA were used as controls. The level of RXRα was reduced over 70% in transfected with RXRα siRNA compared with the scramble siRNA transfected control cells (data not shown). As expected, the DW22 and Bexarotene–mediated viability inhibitions of both cell lines were attenuated by silence of RXRα, whereas there was no effect of RXRα silence on proliferation inhibitory ability of SAHA (Figure 5A). In addition, similar to untransfected HUVEC cells, the DW22 treatment inhibited migration, invasion, and tube formation in scramble siRNA–transfected control HUVEC cells (Figure 5B, and 5D). The DW22–mediated inhibition of HUVECs migration, invasion and tube formation was partly weakened by silence of RXRα. These results confirmed that the DW22–mediated inhibition of cell proliferation, migration, invasion and tube formation was partly dependent on RXRα activation.

Next, we assessed the effect of RXRα silence on DW22–induced cell differentiation and apoptosis. As shown in Figure 5E, DW22–induced differentiation of HL60 cells was almost completely blocked by silence of RXRα, suggesting RXR activation dominantly contributes to DW22-induced cell differentiation. Furthermore, FACS data showed that the DW22–induced apoptosis of A549 and MDA-MB-435 cells was partly attenuated by silence of RXRα (see Figure 5F), indicating RXRα activation also is involved into the DW22–induced apoptosis.

### DW22 retards tumor growth through inhibiting proliferation, angiogenesis and promoting cell apoptosis *in vivo*

**Figure 6:**
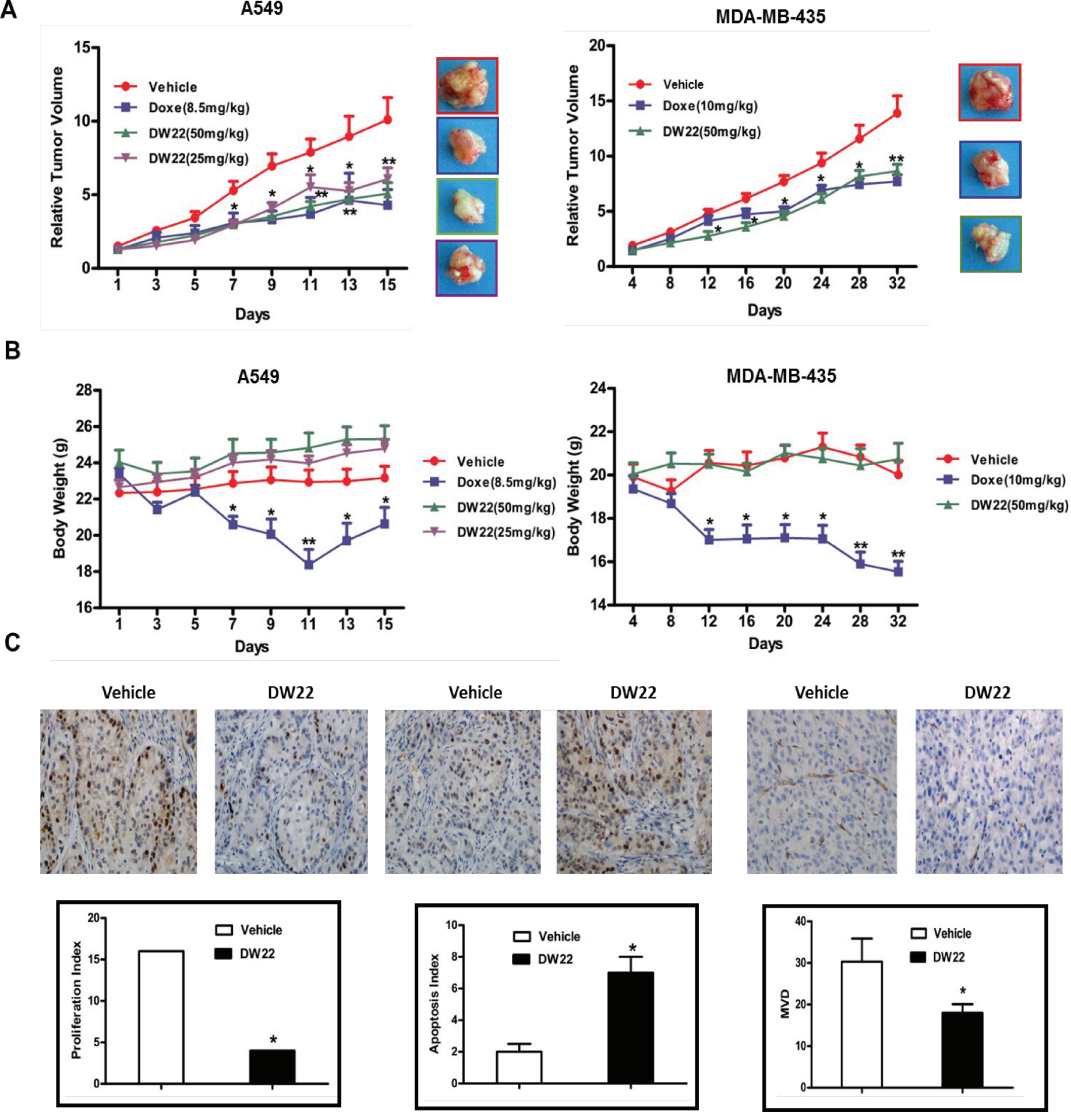
The effects of DW22 on tumor growth, body weight, and the expressions of tumor-related biomarkers *in vivo* **(A)** The effects of relative tumor volume of A549 and MDA-MB-435 xenograft mouse treatmented with DW22 at described concentrations i.v.. **(B)** The effects of body weight of A549 and MDA-MB-435 xenograft mouse treated with DW22 at described concentrations i.v.. **(C)** The effects of tumor-related biomarkers (Ki67, Tunel, and CD31) of MDA-MB-435 xenograft mouse treatmented with DW22 at 50 mg/kg i.v. were measured by immunohistochemistry. All error bars are s.e.m. *p < 0.05 compare with control group, **p < 0.01 compare with control group, ***p < 0.005 compare with control group.

Prompted by the *in vitro* data supporting a potential multiple anti-tumor activity of DW22, we examined the *in vivo* efficacy of DW22 on the growth of A549 and MDA-MB-435 xenograft models, which are demonstrated to be responsive to DW22 by the above *in vitro* experiments. Docetaxel, a first line anti-cancer drug for lung cancer and breast cancer, was used as control and for comparable. As shown in Figure 6A, DW22 could significantly inhibit tumor growth in a dose-dependent manner with the maximal inhibition rate 53% which is comparable to docetaxel (57%) in A549 xenografts. Similar data was obtained from MDA-MB-435 xenografts. Additionally, DW22 did not cause reduced body weight of the host mice (Figure 6B) or other side effects such as hair loss, mortality, and lethargy. In contrast to DW22, docetaxel treatment resulted in an obviously decrease of body weight in both xenograft models (23.1% decrease in A549 xenografts and 22.3% decrease in MDA-MB-435 xenografts).

To confirm the action mechanisms of DW22, we next examined Ki67 staining index for cellular proliferation, terminal deoxynucleotidyl transferase–mediated dUTP nick end labeling (TUNEL) index for apoptosis, and CD31 staining for newly formed blood vessels using MDA-MB-435 xenograft tumor tissues. Treatment by DW22 caused significant decrease of Ki-67 index and microvessel density, and obviously prompted apoptosis *in vivo* (Figure 6C). These data confirmed that the *in vivo* inhibitory effects of DW22 on tumor growth were mediated by inhibition of cell proliferation and angiogenesis, and induction of apoptosis.

## DISCUSSION

RXR and HDAC are considered as important anti-tumor targets, respectively [6, 11]. However, whether combining the two targets could become a novel anti-tumor approach is still not known. In theory, hyperacetylation of histone proteins induced by HDAC inhibitors could increase the accessibility of DNA within chromatin, loosen gene promoter and consequently potentiate the anticancer activities of RXR agonist. Therefore, it is possible dual targeting of RXR and HDAC would exhibit an enhanced antitumor efficiency. In present study, we demonstrate that DW22 effectively targeted RXR/HDAC simultaneously and inhibited tumor cells in multiple aspects. In xenograft tumor models, intravenous administration of DW22 decreased Ki67 and CD31 in tumor tissue, induced apoptosis, and significantly inhibited tumor growth compared to control animals.

DW22 was identified as a dual targeting compound in our previous report [17]. However, the detail mechanism of action of DW22 on RXR and HDAC is not clear. As for activating of RXR, we observed that, similar to Bexarotene, DW22 have no effect on the expression of RXR, but result in an enhanced binding of the RXRα to coactivator peptide PGC1α, which suggesting that elevating RXR binding ability might be the underlying mechanism of DW22 for activating RXR. Our results showed that DW22 could decrease the expression of HDAC1, demonstrating that the downregulation of HDAC might be potential mechanism of DW22 for inhibiting HDAC activity. This molecular mechanism is consistent to SAHA [25], but not to FK228 [26]. Taken together, based on this data, we illustrated that DW22 was a novel dual targeting compound shared the common properties with Bexarotene and SAHA.

RXR is demonstrated to be associated with cell growth, apoptosis and differentiation by modulating the transcriptional activity of retinoid receptor target genes [6]. Our results indicated that DW22 could inhibit cell growth and induce cell apoptosis and differentiation, indicating that DW22 maintains RXR-related biological functions. HDAC has been documented to regulating the expression of genes linked to the inhibition of cell growth, induction of cell differentiation, promotion of apoptosis and suppression of invasion and angiogenesis [12]. Here, the obtained data showed that DW22 blocked cell cycle by downregulating of p21, and inhibited cell invasion and angiogenesis through decreasing expression of VEGF, which verified the nature of DW22 as a HDAC inhibitor. In conclusion, the pleiotropic antitumor activities of DW22 demonstrate dual targeting of RXR and HDAC is a feasible antitumor approach.

Although there exist high differences in biological characteristics between RXR and HDAC, they possess several overlapped biological activities, such as regulation of cell growth and differentiation [6, 12]. Our results revealed that DW22-caused cell growth arrest and apoptosis was partly attenuated, suggesting RXR activation and HDAC inhibition collectively contribute to these events. In addition, the data showed that DW22-induced cell differentiation was completely inhibited by RXR known-down, indicating RXR activation, but not HDAC inhibition, mediates DW22-induced cell differentiation. Interestingly, we also found that DW22-caused inhibition of cell migration, invasion and angiogenesis were partly weakened by RXR knock-down, demonstrating RXR activation also is involved into DW22-mediated inhibitions of cell migration, invasion and angiogenesis. In fact, the RXR agonists, such as Bexarotene, have been reported to inhibit angiogenesis and metastasis [27]. Thus, it is possible that DW22, as a novel RXR agonist, suppress cell migration, invasion and angiogenesis by activating RXR partially.

The significant finding of this study is the co-expression of RXR and HDAC in human lung and breast cancer tissues and cell lines. In addition, it was unexpected that RXR-α was accumulated in the cytoplasm in the most of tumor tissues, suggesting the inactivated RXR is widely presented in the breast and lung cancer. The fact that both RXR and HDAC are extensively expressed in the breast and lung cancer enhances the possibility of success of the proposed tumor therapeutic approach by simultaneous targeting of RXR and HDAC.

**Figure 7:**
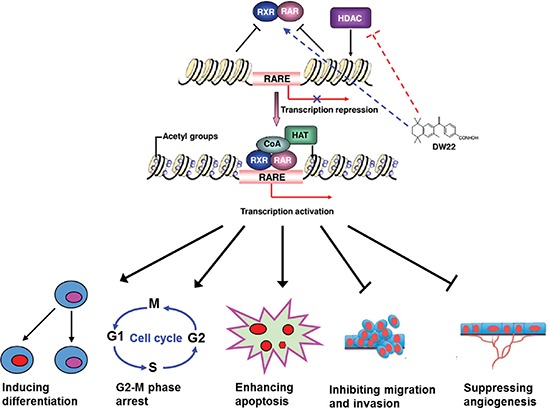
Schematic form of the proposed mechanisms of the effect of DW22 on cancer cells DW22 exerts its anticancer activity on cancer cells by activating RXR and inhibiting HDAC, and synergistically results in the activation of their target gene, subsequently leads to the induction of cell differentiation and apoptosis, the inhibition of cell growth, and the suppression of cell invasion and angiogenesis. HAT(histone acetyltransferase); CoA(Acetyl-CoA); RARE(RAR/RXR binding element).

In summary, this study firstly demonstrates that dual targeting of RXR and HDAC using DW22 possesses pleiotropic antitumor activities *in vitro* and *in vivo* (Figure 7), providing a sound scientific base for developing this novel approach for treatment cancer.

## MATERIALS AND METHODS

### Tumor tissue from patients and immunohistochemistry

Tissues from patients with breast cancer or lung cancer were retrospectively identified from the surgical pathology files of Wuhan General Hospital of Guangzhou Command (Wuhan, PR, China). Pathology slides were analyzed according to tumor size, histologic type, and presence of nodal metastasis. Ethical oversight and approval was obtained from the Institutional Review Board of Wuhan General Hospital of Guangzhou Command. The clinicopathologic features of these patients have been summarized in Supplementary Table 1 and 2. A tissue microarray (TMA) was constructed (in collaboration with the Shanghai Biochip Company Ltd.) as described previously [28]. Following antigen retrieval and blocking, TMA sections (4 mm) were immunostained using antibodies against RXR-α (1:100 dilution; Santa Cruz Biotechnology) and HDAC1 (1:200 dilution; Abcam) with detection using the avidin–biotin complex method (DAKO) visualized by 3,30–diaminobenzidine (DAB). Slides were lightly counterstained with hematoxylin. The evaluation of both the intensity of immunohistochemical staining and the proportion of positively stained epithelial cells was previously described [29]. A detailed description of the analysis is provided in the Supplementary Materials and Methods.

### Compounds and reagents

DW22, with purity more than 98%, was synthesized in Medicine Chemistry Laboratory at Shenyang Pharmaceutical University (see Figure 2A). Bexarotene and SAHA were obtained from Sigma, USA. These agents were dissolved in DMSO to 100 mM and stored at −20°C. Before treatment, the stock solution is diluted to different concentrations. The final concentration of DMSO in cultures is 0.1% (v/v) or less. MTT (3-(4,5-dimethylthiazol-2-yl)-2,5-diphenyl tetrazolium bromide) and Propidium idodide (PI) were purchased from Sigma, USA. The primary antibodies against RXRα, HDAC1, Histone 3, Histone 4, p21 ^WAF1/CIP1^, PARP, Caspase-3, KLF4, VEGF, and β-actin were got from Cell Signaling Technology (Danvers, MA). The primary antibodies against Ki67, CD31, specific RXRα siRNA, and control siRNA duplexes were obtained from Santa Cruz Biotechnology (Santa Cruz, CA). The primary antibodies against acetylated Histone 3 and acetylated Histone 4 were purchased from Millipore, USA. The HDAC1 Silencer Select Validated siRNA was got from Life technologies, USA.

### Cell lines and cell culture

Human lung cancer cell lines A549, NCI-H460, NCI-H1299, human breast cancer cell lines MDA-MB-435, MDA-MB-436, MCF7, MDA-MB-231, human leukemia cell line HL-60, and human umbelical vein cells (HUVEC) were obtained from the American Type Culture Collection (Manassas, VA). Human lung cancer cell line LTEP-α2 was provided from National Center for Medical Culture Collection (Shanghai, CHN). These cancer cells were routinely cultured in RPMI-1640 or MEM medium supplemented with 10% fetal bovine serum (FBS) and maintained at 37°C in a humidified incubator with 5% CO_2_. HUVEC cells were maintained as monolayer in MCDB131 medium supplemented with 20% (v/v) fetal bovine serum (FBS), 1% (v/v) L-glutammine, 5 units/mL heparin, and 50 mg/mL endothelial cell growth factor (ECGF) using culture flasks or plates precoated with 1% (v/v) gelatin. All cell lines used were between passages 3 and 8 for each experiment and were demonstrated to be free of mycoplasma using Mycoplasma PCR Detection Kit (Sigma, USA).

### Cell viability assay

The *in vitro* cell viability effects of DW22 were determined by MTT assay. The cells (1 × 10^5^ cells/ml) were seeded into 96-well culture plates. After overnight incubation, the cells were treated with various concentrations of agents for 12, 36 or 48 h. Then 10 μl MTT solution (2.5 mg/ml in PBS) was added to each well, and the plates were incubated for an additional 4 h at 37°C. After centrifugation (2500 rpm, 10 min), the medium with MTT was aspirated, followed by the addition of 100 μl DMSO. The optical density of each well was measured at 570 nm with a Biotek Synergy^TM^ HT Reader.

### RXRα coactivator recruitment assay

Time-resolved fluorescence resonance energy transfer (TR-FRET) was performed with the LanthaScreen TR-FRET RXRα coactivator assays according to the instructions of the manufacturer (Invitrogen). Incubation time was 2 hours in this study. The 520/490 TR-FRET ratio was measured with Spectramax Paradigm instrument (Molecular Devices, USA) with instrument settings as described in the manufacturer's instructions for LanthaScreen assays. The following peptides were used for the coactivator recruitment assay: PGC1α, EAEEPSLLKKLLLAPANTQ.

### RXR agonist activity assay

The A549 and MDA-MB-435 cells were cotransfected with CRBPII-tk-luc (a gift from makishima.makoto, Nihon University School of Medicine, JAPAN), pCMX-hRXR, and pRL Renilla Luciferase Reporter Vector (Promega Corporation). The ratio of these three plasmids is 10: 3: 2. Five hours after transfection, the cells were treated with different concentrations of DW-22 and Bexarotene. After 48 h transfection, the RXR activity was detected with Dual-Luciferase Reporter Assay System (Promega Corporation). RXR activity is presented as the means ± SD of three determinants.

### HDAC whole-cell assay

The A549, NCI-H460, MDA-MB-436 and MDA-MB-435 cells were treated with different concentrations of DW22 and SAHA for 24 h before assays. Proteins were isolated by using cell lysis buffer (Beyontime, CHN). The protein concentration was measured by BCA protein assay (Beyontime, CHN). The HDAC activity was measured with a HDAC fluorescent activity assay kit (Biovison, USA). In brief, cell lysates were incubated with the HDAC substrate Boc-Lys(Ac)-pNA (10 mM) in HDAC assay buffer. After 90 min at 37°C, reactions were stopped by adding 10 μL of Lysine Developer and further incubated for 30 min at 37°C. Absorbance was measured using a microplate reader (Molecular Devices). HDAC activity is presented as the means ± SD of three determinants.

### Clonogenicity assay

The A549 and MDA-MB-435 cells were treated with different concentrations of DW22, Bexarotene, and SAHA. Then the cells were incubated for an additional 7 days. Treatments were carried out in triplicate. The colonies obtained were formalin fixed and stained with hematoxylin. The colonies were counted and compared with untreated cells.

### NBT reduction assay

The HL60 cells were treated with different concentrations of DW22 and Bexarotene for 4 days. Cells were incubated in RPMI1640 (10% FBS) and an equal volume of phosphate-buffer saline containing 0.2 w/w% NBT (Sigma) and 12-O-tetradecanoylphorbol-13-acetate (TPA, 200 ng/mL) in a humidified atmosphere of 5% CO_2_ at 37°C for 30 min. The rate of cell differentiation was calculated by the percentage of cells containing blue–black formazan using more than 200 cells. Average of at least three results for each assay was calculated.

### Quantitative PCR analysis

Total RNA was isolated from A549 cells using RNeasy Mini Kit (Qiagen) as described in the product insert. The RNA was reverse RevertAid First Strand cDNA Synthesis Kit (Thermo) and PCR was done using iQ SYBR Green Supermix and the CFX96 Real-Time PCR Detection System (Bio-Rad). Primers used were glyceraldehyde-3-phosphate dehydrogenase (*GAPDH*). Reverse primer 5′-CCC TCA ACG ACC ACT TTG TCA-3′ and forward primer 5′-TTC CTC TTG TGC TCT TGC TGG-3′; *p21^WAF-1^* reverse primer 5′-GTC CAG CGA CCT TCC TCA TCCA-3′ and forward primer 5′-CCA TAG CCT CTA CTG CCA CCA TC-3′.

### Chromatin immuno-precipitation assay (ChIP)

Using ChIP Assay Kit (Beyontime, CHN), A549 cells were prepared for the ChIP assay performed as the instructions of the manufacturer. Anti-H3Ac antibody was used to immunoprecipitate histones. *p21^WAF-1^* promoter primers were used to carry out PCR from DNA isolated from ChIP experiment. PCR products were analyzed by 2.0% agarose/ethidium bromide gel electrophoresis. The primer pairs used: Forward primer 5′-CGT GGT GGT GGT GAG CTA GA-3′ (*p21^WAF1^* primer 1), Reverse primer 5′-CTG TCT GCA CCT TCG CTC CT-3′ (*p21^WAF1^* primer 1), Forward primer F5′-GGT GTC TAG GTG CTC CAG GT-3′ (*p21^WAF1^* primer 2), Reverse primer 5′-GCA CTC TCC AGG AGG ACA CA-3′ (*p21^WAF1^* primer 2). Data are presented as fold enrichment which was calculated by anti-H3Ac antibody ChIP value (IP/Input, the percentage of input) of DW22/SAHA treated cells relative to ChIP value of DMSO treated cells.

### Flow cytometry analysis

About 1 × 10^6^ A549 and MDA-MB-435 cells were harvested at room temperature after pre-treatment with DW22, Bexarotene, and SAHA for 48 h. The supernatant was removed, and ice-cold 70% ethanol was added. Ethanol-fixed cells were re-suspended in PBS containing 0.1 mg/ml RNase and incubated at 37°C for 30 min. The pelleted cells were suspended in 1.0 ml of 40 μg/ml propidium iodide (PI) and analyzed by using a flow cytometer (Becton Dickinson, San Jose, CA). The cell cycle distribution was estimated according to standard procedures. The percentage of cells in the different cell cycle phases (G0/G1, S, or G2/M phase) was calculated using CELLQuest (Becton Dickinson) software. The cells of sub-G1 peak were considered as apoptosis.

Analyses for apoptosis were also conducted with an Annexin V–FITC Apoptosis Detection Kit (BioVision). Cells (1 × 10^6^) were exposed to DW22 (20 μM) and Bexarotene (20 μM) for 48 h. They were collected by centrifugation and resuspended in 500 μL of 1 × binding buffer. Annexin V–fluorescein isothiocyanate (FITC; 5 μL) and PI (5 μL) were added to the cells. After incubation at room temperature for 5 minutes in the dark, cells were analyzed by FACS using a flow cytometer (Becton Dickinson). Cells that stained Annexin V–FITC only (early apoptosis) were analyzed.

### Western blot analysis

About 1 × 10^7^ cells were gathered after pre-treatment for the indicated time periods as described previously. Western blotting was performed as previously described [30]. Briefly, an equal amount of total protein extracts from cultured cells or tissues were fractionated by 10–15% SDS-PAGE and then electrically transferred onto polyvinylidene difluoride (PVDF) membranes. Mouse or rabbit primary antibodies and appropriate horseradish peroxidase (HRP)-conjugated secondary antibodies were used to detect the designated proteins. The bound secondary antibodies on the PVDF membrane were reacted with ECL detection reagents (Pierce; Rockford, USA) and exposed to X-ray films. Results were normalized to the internal control β-actin.

### Wound-healing migration assay

Wound-healing migration assay was performed as described previously [31]. Briefly, HUVECs were starved to inactivate cell proliferation and then wounded by pipette tips. ECGM containing 0.5% FBS was added with or without 20 ng/ml VEGF and different dilutions of DW22. Images of the cells were taken after 12 h of incubation. Migrated cells were quantified manually. Three independent experiments were performed.

### Real-time cell analysis (RTCA)

The CIM-plate contains 16 modified Boyden chambers, which can be used independently to measure cell migration in real time through 8 μm pores of a polyethylene terephthalate membrane onto gold electrodes on the underside of themembrane using the xCELLigence analyser system (ACEA Biosciences). Experiments were set up according to the manufacturer's instructions with the membrane uncoated (migration). A chemotactic signal for movement was provided by inoculating 30,000–50,000 cells in serum-free medium in the upper chamber and supplying 10% FBS in the lower chamber (10 ng/ml VEGF with the same relevant concentration of drug). Cell index (electrical impedance) was monitored every 5 min for the duration of the experiment. The cell index represents the capacity for cell migration, and the slope of the curve can be related to the migration velocity of tumor cells. The cell index thus reflects the tumor cell's migratory capacity.

### Transwell migration assay

Invasion of HUVECs was assayed using Transwell (Corning Costar) with 6.5 mm diameter polycarbonate filters (8-Am pore size). Briefly, the lower surface of the filter was coated with Matrigel. Fresh M200 medium (1% FBS) containing 20 ng/ml VEGF was placed in the lower wells. The cells were trypsinized and suspended at a final concentration of 1 × 10^6^ cells/ml in M200 containing 1% FBS. Various concentrations of DW22 were given to the cells for 30 min at room temperature before seeding. One hundred microliters of the cell suspension were loaded into each of the upper wells, and the chamber was incubated at 37°C for 12 h. The cells were fixed and stained with Calcein-AM. Non-invasion cells on the upper surface of the filter were removed by wiping with a cotton swab, and chemotaxis was quantified with a high content drug screening system ImageXpress^R^ Micro (Molecular Devices) by counting cells that had migrated to the lower side of the filter.

### Tube formation assay

HUVECs was incubated in M200 containing 2% LSGS (Low Serum Growth Supplement). Various concentrations of DW22 were added to the cells for 30 min at room temperature before seeding. Cells were plated onto the layer of Matrigel at a density of 1.8 × 10^5^ cells per well, followed by the addition of 20 ng/ml VEGF. After 12 h, the cells were fixed and stained with Calcein-AM. The area covered by the tube network was determined using the high content drug screening system ImageXpress^R^ Micro (Molecular Devices) after photographed (400 ×).

### RNA interference of HDAC1 and RXRα

RNA interference of RXRα was done using an HDAC1 or RXRα–targeted short interfering RNA (siRNA; Santa Cruz). A nonspecific scramble siRNA was used as control. For transfection, A549, MDA-MB-435, HL-60 and HUVECs cells (5 × 10^4^) were seeded in 96 or six-well plates and allowed to attach overnight. Cells were transfected with scramble siRNA, RXRα–targeted siRNA(200 pmol/L) or HDAC1- targeted siRNA(90 pmol/L) using OligofectAMINE (Invitrogen) according to the manufacturer's recommendations. The efficiency of siRNA was confirmed by western blot. Twenty-four hours after transfection, the cells were treated with DMSO (control) or different concentrations of DW22 for 12, 24, or 48 h. The cells were collected and processed for analysis of MTT, NBT, FACS, migration, tube formation and immunoblotting as described above.

### Mouse xenograft tumors study

To determine the *in vivo* anti-tumor activity of DW22, viable A549 cells (5 × 10^6^/100 μl PBS per mouse) and MDA-MB-435 cells (1 × 10^7^/100 μl PBS per mouse), as confirmed by trypan blue staining, and then subcutaneously (s.c.) injected into the right flank of 7–8 week old male Balb/c nude mice or SCID mice, respectively. When the average s.c. tumor volume reached 100 mm^3^, the mice were randomly divided into various treatment and control group (5–6 mice per group). Tumor size was measured once every two days with a caliper (calculated volume = shortest diameter^2^ × longest diameter/2). Body weight, diet consumption and tumor size were recorded once every two days. After two or three weeks, the mice were sacrificed and solid tumors were removed for further analysis. This study was performed in strict accordance with the recommendations in the Guide for the Care and Use of Laboratory Animals of the Shenyang Pharmaceutical University. The protocol was approved by the Committee on the Ethics of Animal Experiments of the Shenyang Pharmaceutical University.

### Immunohistochemistry and TUNEL assay

Tissues embedded in paraffin were cut to a section of 4 μm, deparaffinized, and treated with citrate buffer. Then, they were blocked with avidin/biotin for 20 min. The slides were incubated with anti-Ki67 or CD31 for overnight at 4°C. Next, the slides were treated with secondary antibody with horseradish peroxidase goat anti-rabbit for 1–3 h and developed with 3, 3-diaminobenzidine (Sigma-Aldrich). Finally, the slides were counterstained with hematoxylin. Terminal deoxynucleotide transferase-mediated dUTP nick end labeling (TUNEL) system (Roche, Switzerland) was used to detect apoptosis in the tumor sections placed on slides according to the manufacturer's protocol. TUNEL reaction solution was substituted with TdT-free solution for a negative control. Sections were pretreated 10 min with DNase and visualized by diaminobenzidine (DAB) staining. Positive nuclei were identified by brown color.

### Statistical analysis

Differences between experimental groups were evaluated by one-way ANOVA or Turkey's post-hoc test using the SPSS11.5 software package for Windows (SPSS, Chicago, IL). Survival curves were constructed using the Kaplan–Meier method. Statistical significance was based on a *p*-value of 0.05 (*p* < 0.05, two-tailed test).

## SUPPLEMENTARY FIGURE AND TABLES


