# Supplementary material for: Dual targeting of retinoid X receptor and histone deacetylase with DW22 as a novel antitumor approach
Source: Oncotarget. 2015 Feb 5;6(12):9740–55. doi: 10.18632/oncotarget.3149 (PMC4496394; doi:10.18632/oncotarget.3149)
Supplement: Supplementary file 1 [file oncotarget-06-9740-s001.pdf]

SUPPLEMENTARY MATERIALS AND METHODS

Evaluation of Immunohistochemistry results

The previous described scoring method (1, 2) was used to evaluate both the intensity of immunohistochemical staining and the proportion of the stained epithelial cells. The staining intensity was classified as follows: 1, weak; 2, moderate; and 3, strong. Positive cells were quantified as a percentage of the total number of epithelial cells, and were assigned to one of five categories: 0, < 5%; 1, 5–25%; 2, 26–50%; 3, 51–75%; and 4, > 75%. The percentage of positive tumor cells and the staining intensity were then multiplied in order to generate the immunoreactive score (IS) for each of the tumor specimens. A sample is defined as RXRα or HDAC1 + if it has an immunoreactive score ≥ 2. Each lesion was separately examined and scored by two pathologists, and discrepancies were discussed to obtain a consensus. An IS value of more than 2 was regarded as a high level of expression.

REFERENCES

1. Sinicrope FA, Ruan SB, Cleary KR, Stephens LC, Lee JJ, Levin B. bcl-2 and p53 oncoprotein expression during colorectal tumorigenesis. Cancer Res. 1995; 55:237–41.
2. Wang LH, Kim SH, Lee JH, Choi YL, Kim YC, Park TS, Hong YC, Wu CF, Shin YK. Inactivation of SMAD4 Tumor Suppressor Gene During Gastric Carcinoma Progression. Clin Cancer Res. 2007; 13:102–10.

SUPPLEMENTARY FIGURES AND TABLES

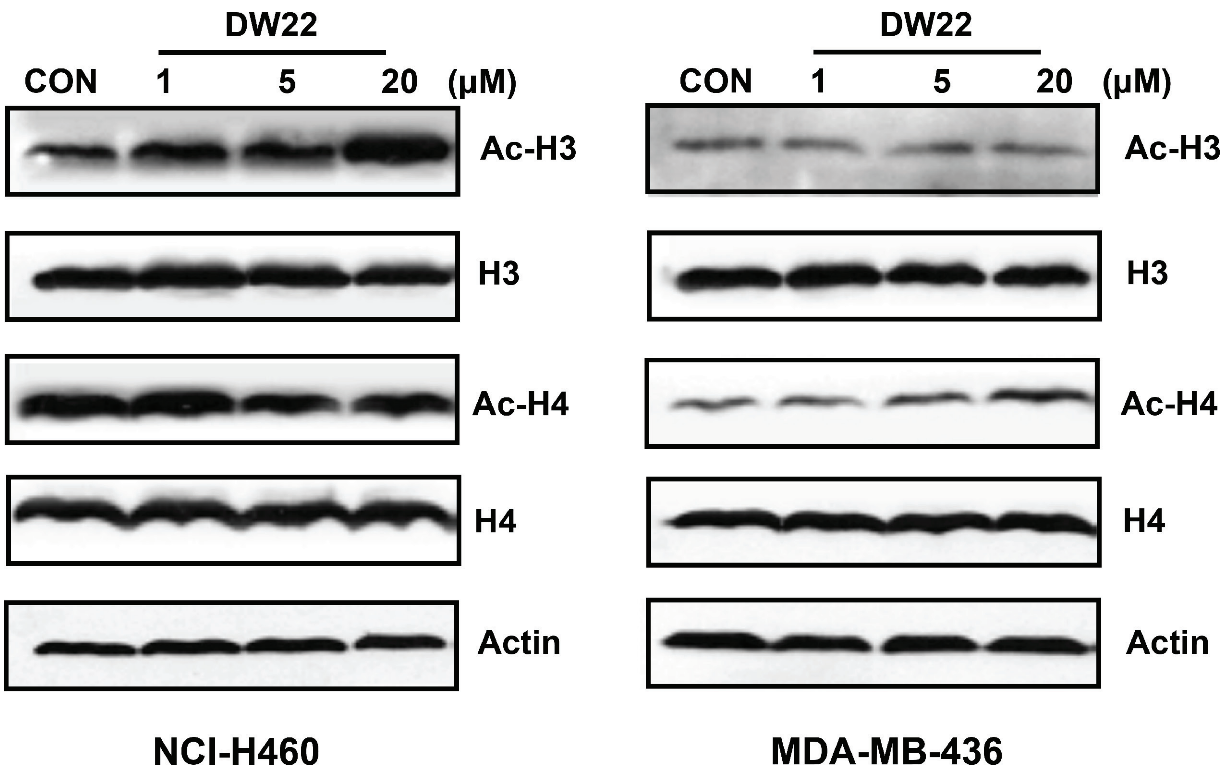

Supplementary Figure 1: The effect of DW22 on Ac-H3 and Ac-H4 in NCI-H460 and MAD-MB-436 cells. Acetylated histone-3(Ac-H3, MW 17 kD), histone-3(H3, MW 17 kD), acetylated histone-4(Ac-H4, MW 10 kD), and histone-4(H4, MW 11 kD), were measured in NCI-H460 and MAD-MB-436 cell lines after DW22 (1, 5, 20 μM) treated for 48 h. β-actin expression was used as a loading control.

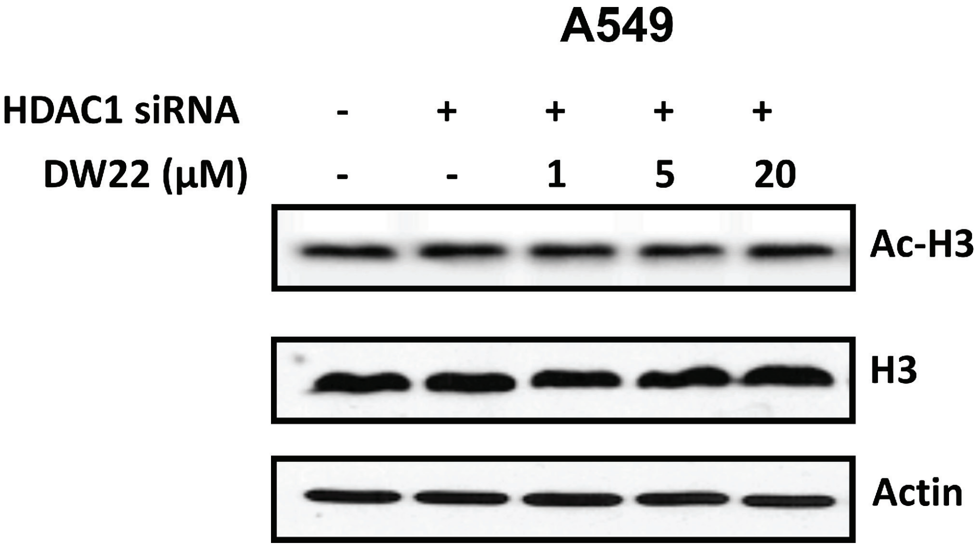

Supplementary Figure 2: The effect of knockdown of HDAC1 on DW22 induced up-regulation of Ac-H3. Acetylated histone-3(Ac-H3, MW 17 kD) and histone-3(H3, MW 17 kD) were measured in HDAC1 silenced A549 cells after DW22 (1, 5, 20 μM) treated for 48 h. HDAC1 was silenced by specific siRNA (90 pM). β-actin expression was used as a loading control.

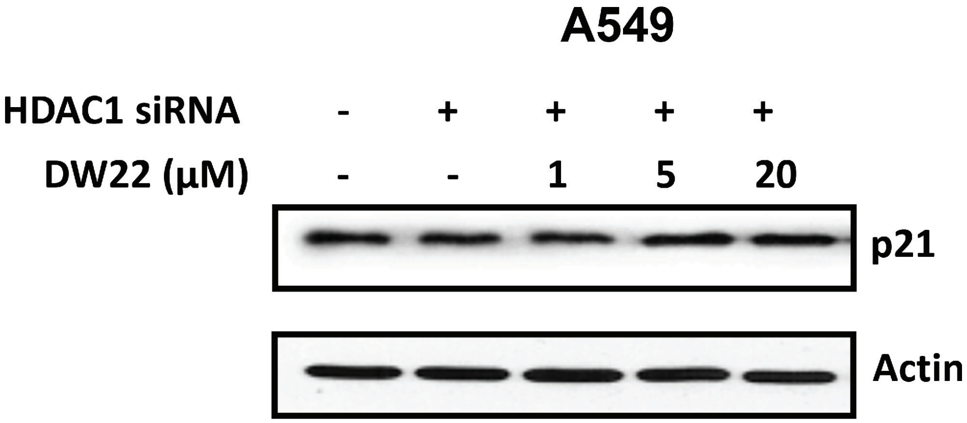

Supplementary Figure 3: The effect of knockdown of HDAC1 on DW22 induced up-regulation of p21. The p21 protein was measured in HDAC1 silenced A549 cell lines after DW22 (1, 5, 20 μM) treated for 48 h. HDAC1 was silenced by specific siRNA (90 pM). β-actin expression was used as a loading control.

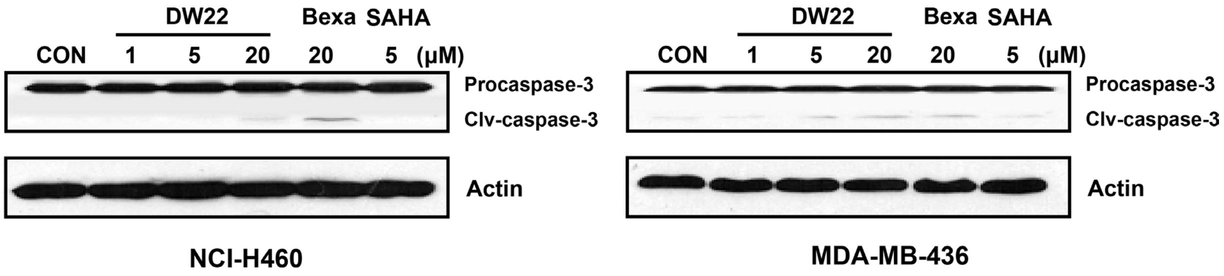

Supplementary Figure 4: The effect of DW22 on caspase-3 activation in NCI-H460 and MAD-MB-436 cells. The caspase-3 and clv-caspase-3 protein were measured in NCI-H460 and MAD-MB-436 cell lines after DW22 (1, 5, 20 μM) treated for 48 h. β-actin expression was used as a loading control. Caspase-3, MW 32 kD; clv-caspase-3, MW 17 kD.

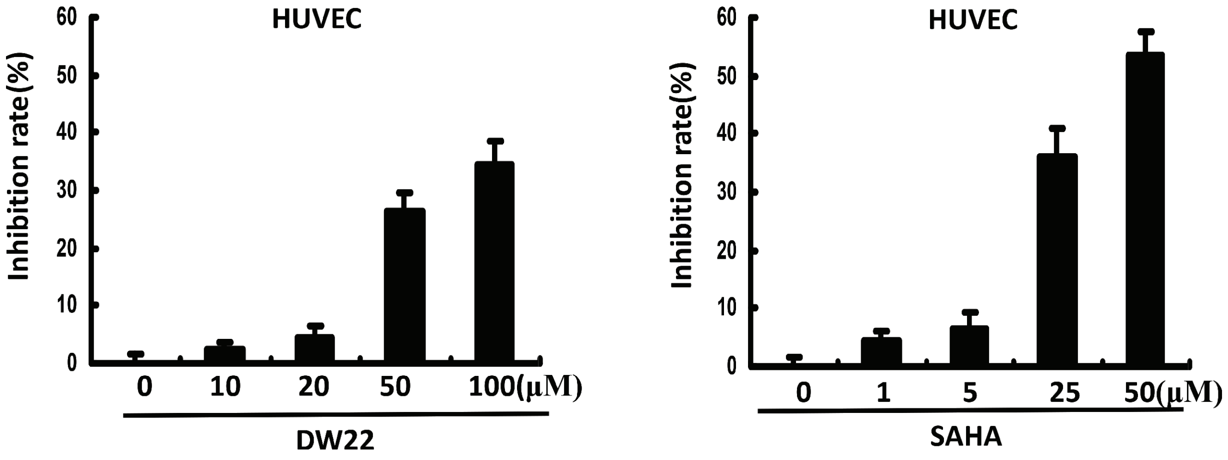

Supplementary Figure 5: The effect of DW22 on viability of HUVECs. The cell survival of HUVECs with different concentrations 10, 20, 50, and 100 μM of DW22 treatment (12 h) was detected using MTT assay.

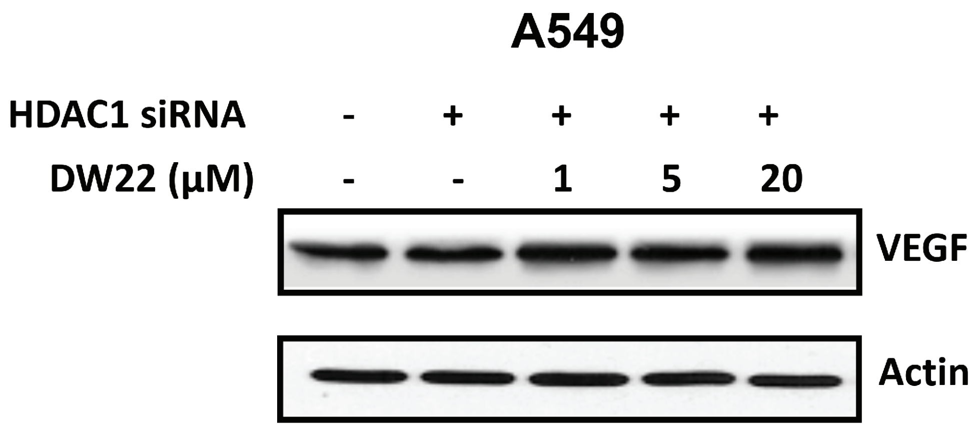

Supplementary Figure 6: The effect of knockdown of HDAC1 on DW22 caused down-regulation of VEGF. The VEGF protein was measured in HDAC1 silenced A549 cell lines after DW22 (1, 5, 20 μM) treated for 48 h. HDAC1 was silenced by specific siRNA (90 pM). β-actin expression was used as a loading control. VEGF, MW 43 kD.

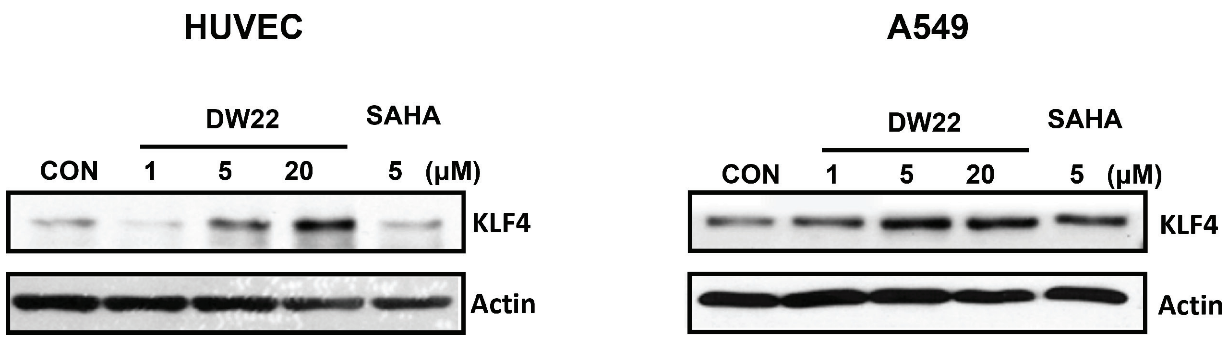

Supplementary Figure 7: The effect of DW22 on KLF4 expression in HUVEC and A549 cells. The KLF4 protein was measured by western blotting in HUVEC and A549 cell lines after DW22 (1, 5, 20 μM) treated for 24 h. β-actin expression was used as a loading control. KLF4, MW 62 kD.

Supplementary Table 1: Clinicopathological parameters in breast cancer patients

Variable	N
**Age**	
< 50 years	47
≥ 50 years	59
**Tumor size**	
T1	41
T2	55
T3	10
**Node metastasis**	
Negative	56
Positive	50
**Histologic type**	
invasive ductal carcinoma	106
**pTNM staging**	
0-I	32
II	49
III	25

Supplementary Table 2: Clinicopathological parameters in lung cancer patients

Variable	N
**Age**	
< 65 years	46
≥ 65 years	56
**Tumor size**	
< 3.0 cm	43
≥ 3.0 cm	59
**Node metastasis**	
Negative	61
Positive	41
**Histologic type**	
Squamous carcinoma	32
Adenocarcinoma	70
**pTNM staging**	
0-I	40
II	35
III	27
